# The Early Positive Approaches to Support (E-PAtS) study: study protocol for a feasibility cluster randomised controlled trial of a group programme (E-PAtS) for family caregivers of young children with intellectual disability

**DOI:** 10.1186/s40814-020-00689-9

**Published:** 2020-10-02

**Authors:** Elinor Coulman, Richard Hastings, Nick Gore, David Gillespie, Rachel McNamara, Stavros Petrou, Jeremy Segrott, Jill Bradshaw, Kerry Hood, Andrew Jahoda, Geoff Lindsay, Fiona Lugg-Widger, Michael Robling, Jacqui Shurlock, Vaso Totsika

**Affiliations:** 1 Centre for Trials Research, Cardiff University, Neuadd Meirionnydd, Heath Park, Cardiff, CF14 4YS UK; 2 Centre for Educational Development Appraisal and Research, University of Warwick, Coventry, CV4 7AL UK; 3 Tizard Centre, University of Kent, Cornwallis North East, Canterbury, CT2 7NF UK; 4 Warwick Clinical Trials Unit, University of Warwick, Coventry, CV4 7AL UK; 5 DECIPHer Centre Cardiff University, 1-3 Museum Place, Cardiff, CF10 3BD UK; 6 Institute of Health and Wellbeing, University of Glasgow, G12 8RZ, Glasgow, UK; 7 The Challenging Behaviour Foundation, The Old Courthouse, New Road Avenue, Chatham, Kent, ME4 6BE UK; 8 Division of Psychiatry, Faculty of Brain Sciences, University College London, Wing B, Maple House, Tottenham Court Road, London, UK

**Keywords:** Intellectual disability, Learning disability, Support, Randomised controlled trial, Feasibility, Early Positive Approaches to Support (E-PAtS), Mental wellbeing

## Abstract

**Background:**

Children with intellectual disability have an IQ < 70, associated deficits in adaptive skills and are at increased risk of having clinically concerning levels of behaviour problems. In addition, parents of children with intellectual disability are likely to report high levels of mental health and other psychological problems. The Early Positive Approaches to Support (E-PAtS) programme for family caregivers of young children (5 years and under) with intellectual and developmental disabilities is a group-based intervention which aims to enhance parental psychosocial wellbeing and service access and support positive development for children. The aim of this study is to assess the feasibility of delivering E-PAtS to family caregivers of children with intellectual disability by community parenting support service provider organisations. The study will inform a potential, definitive RCT of the effectiveness and cost-effectiveness of E-PAtS.

**Methods:**

This study is a feasibility cluster randomised controlled trial, with embedded process evaluation. Up to 2 family caregivers will be recruited from 64 families with a child (18 months to 5 years) with intellectual disability at research sites in the UK. Participating families will be allocated to intervention: control on a 1:1 basis; intervention families will be offered the E-PAtS programme immediately, continuing to receive usual practice, and control participants will be offered the opportunity to attend the E-PAtS programme at the end of the follow-up period and will continue to receive usual practice. Data will be collected at baseline, 3 months post-randomisation and 12 months post-randomisation. The primary aim is to assess feasibility via the assessment of: recruitment of service provider organisations; participant recruitment; randomisation; retention; intervention adherence; intervention fidelity and the views of participants, intervention facilitators and service provider organisations regarding intervention delivery and study processes. The secondary aim is preliminary evaluation of a range of established outcome measures for individual family members, subsystem relationships and overall family functioning, plus additional health economic outcomes for inclusion in a future definitive trial.

**Discussion:**

The results of this study will inform a potential future definitive trial, to evaluate the effectiveness and cost-effectiveness of the E-PAtS intervention to improve parental psychosocial wellbeing. Such a trial would have significant scientific impact internationally in the intellectual disability field.

**Trial registration:**

ISRCTN70419473

## Background

Children with intellectual disability (ID) are defined as having an IQ < 70. They have associated deficits in adaptive skills, with impairments emerging in the ‘developmental period’—typically considered to be before age 18 years. Just over 2% of children in England have been identified by local authorities/schools as having ID, according to data by the UK Learning Disability Observatory [1], and although prevalence varies slightly with socio-economic factors, it appears broadly similar across the UK.

UK population-based data have shown that children with ID are 4–5 times more likely to have a diagnosable mental health disorder [2] compared to other UK children. In addition, high proportions (60–80%) of children with ID in population-based samples have clinically concerning levels of behaviour problems (including hyperactivity and conduct problems) [3]. These health inequalities for children with ID emerge by the time the child with ID is 3–5 years of age at the latest [4].

Furthermore, longitudinal studies suggest that increased behavioural and emotional problems in the child with ID leads to deterioration in parental wellbeing over time, and vice versa [5, 6]. Data from UK population-based research show that parents, especially mothers, of children with ID are 2–3 times more likely to report elevated or clinically concerning levels of mental health and other psychological problems when compared to parents who do not have a child with ID [3]. Population-based data for the UK and other countries suggest that rates of mental health problems at a level of clinical concern range from between approximately one third and one half of this population of parents [5]. Thus, parents of young children with ID represent a high-risk population in terms of parental psychosocial (ill) health.

Moving beyond the dyadic association between the wellbeing of a child with ID and a parent, family research studies applying family systems theory [7, 8] show findings that reflect similar data in other family members. For example, the psychological problems of the child with ID can negatively affect sibling wellbeing, as well as that of parents [5]. In addition, parental relationship problems, parent-child relationships, sibling relationships and overall family functioning may all be adversely affected in families of children with ID [5]. Parental wellbeing in families of children with ID may also be more strongly (or at least as strongly) associated with their partner’s wellbeing than with their child’s [5].

Furthermore, access to specialised supports is also a challenge for families of children with ID. For example, less than 30% of parents of children with ID who also had a diagnosable mental health problem had access to mental health services in the preceding 12 months [9]. Thus, children with ID and their parents face significant health inequalities and potential problems accessing appropriate support.

Given the research evidence, interventions are needed that target both parental wellbeing and child health outcomes, especially taking into account the very high levels of behaviour problems in young children with ID. Furthermore, parenting behaviours and parent-child relationship factors have been shown in longitudinal research studies to affect the short- to medium-term course and severity of behaviour problems in children with ID [5, 10]. Thus, interventions that target both parenting practices/strategies and parent-child relationships could have significant potential to support families of young children with ID.

Recent systematic reviews of parenting interventions for parents of children with ID have been conducted by NICE to inform the Mental Health Problems in People with Learning Disability [11]. Although 15 RCTs of parenting programmes involving parents of children with ID were reviewed, parent wellbeing was not the focus of any of these programmes, the programmes were not developed specifically for parents of children with ID but were adapted from mainstream parenting programmes [12], and the programmes were not targeted at families of young children specifically. The single exception was a RCT of an individual-family delivered positive behavioural support intervention for young children with ID and severe behaviour problems [13], comparing the intervention alone to a version including a parent optimism component. Thus, NICE found no evidence of group parenting programmes designed specifically for parents of young children with ID, without a specific focus on a problem related to the child (e.g., severe behaviour problems) and with the explicit aim of improving parent psychosocial wellbeing, therefore demonstrating a gap in the evidence base.

E-PAtS directly addresses the gap in both the availability of suitable group parenting programmes and in the evidence base. The E-PAtS bespoke family caregiver programme has been co-produced by family caregivers and professionals and is specifically informed by existing ID research evidence from children with ID and their families [14], in addition to developmental system approaches to early intervention [15]. E-PAtS is also routinely co-facilitated by a family caregiver and professional working in partnership as an 8 session group programme, suitable for all families of young children with ID, to support caregiver wellbeing, service access and positive development for children.

Parenting programmes for families of children with ID are likely to be a priority for UK services for several decades. For example, in England, ID services across the NHS, local authorities and the for-profit and third sector are undergoing considerable change as a result of the government’s Transforming Care programme. The service model from the Transforming Care programme [16] identifies early intervention/early support, and support and skills training for parents as a part of a regional/community response to better services for families of children with ID. In Scotland, parenting interventions are also a priority and are seen as a key way to improve the life chances of disadvantaged groups, including children with ID. The Scottish Government has proposed a coordinated parenting strategy across statutory and third sector organisations, with partners from the third sector taking a lead in delivering parenting interventions [17]. A feasibility study for E-PAtS, with the potential for a later large scale RCT evaluation, would therefore make a significant contribution to providing UK evidence and informing on-going policy.

Apart from direct relevance to UK policy and practice, evidence from a robust programme of research on E-PAtS has the potential for substantial international scientific and policy impact. First, existing RCTs of group parenting programmes with parents of children with ID involve small samples only. Second, existing intervention studies have included, and thus measured outcomes typically for, only one parent in each family. Thus, effectiveness for fathers or for the non-included parent in the family remains unknown. Third, data analysis of existing group intervention RCTs has often failed to take account of the nested nature of the data (i.e. failing to account for parents clustered within the parenting groups that they attend). This study, as a potential to inform a large definitive RCT, may be a stepping stone to directly address these points and thus have significant scientific impact internationally in the ID field.

## Methods/design

### Objectives/aim

The aim of the feasibility RCT is to assess the feasibility of delivering E-PAtS to family caregivers of children with ID by community parenting support service provider organisations. The study will aim to contribute to the evidence base on improving outcomes for children with ID and their family caregivers. Importantly, the study will inform a potential, definitive RCT of the effectiveness and cost-effectiveness of E-PAtS.

The study’s primary objectives are to assess the following:

- The feasibility of recruiting eligible participants to the study and the most effective recruitment pathways to identify families of young children with ID.
- The feasibility of recruiting suitable intervention providers and facilitators to deliver the E-PAtS intervention.
- Recruitment rates and retention through 3 months and 12 months post-randomisation follow-up data collection.
- The acceptability of study processes, including randomisation, to both service provider organisations, facilitators and family caregivers through qualitative interviews.
- The acceptability of intervention delivery to both service provider organisations, facilitators and family caregivers through qualitative interviews.
- Adherence to the intervention, reach and fidelity of implementation of the E-PAtS intervention through attendance records, evaluation of session recordings and participant/facilitator qualitative interviews.
- Usual practice in this setting and use of services/support by intervention and control participants.
- The feasibility and acceptability of proposed outcome measures for a definitive trial, including resource use and health-related quality of life data, as methods to measure effectiveness of the intervention and to conduct an embedded health economic evaluation within a definitive RCT.
- Acceptability of collecting and analysing routinely collected data within a definitive RCT.
- Service provider organisation willingness to participate in a definitive trial.

### Study design

The study is a 2-arm, cluster (family caregivers in families) randomised controlled trial, with 1:1 randomisation using randomly permuted blocks, stratified by study site and choice of either study pathway A (control participants to be offered E-PAtS on a waitlist) or pathway B (control participants not offered E-PAtS). Participants will be recruited, asked to select study pathway and randomised. Intervention participants will be offered E-PAtS immediately, and all participants will continue to have access to usual support and advice services provided.

### Study setting

The study will take place in up to 4 study sites, defined as geographical areas where service provider organisations offer support services to parents provider organisations.

### Site selection

Service provider organisations will be selected as sites for the E-PAtS feasibility study if they fulfil the following selection criteria:

1. Service provider organisations are prepared to refer a sufficient number of potential participants/families to the study team.
2. Service provider organisations are prepared to deliver up to 2 E-PAtS courses at two periods throughout the study: (1) immediately following randomisation and (2) following data collection 12 months post-randomisation.

### Participant selection

Families will be referred to the study team by service provider organisations in their local area following a flexible multi-point recruitment method including via: established referral routes, local and national charitable support organisations, local authority services, special schools and nurseries, after school/weekend services for children with special educational needs and disabilities, parent/family support groups, social media, advertising in the media in local areas, and self-referral.

The strategy is aimed to be flexible and collaborative, and information will be gathered regarding the most effective participant-identification processes to inform a definitive trial.

All potential participants will have been provided with a participant information sheet and will have confirmed interest in participating in the study either directly with the service provider organisation or by returning a completed reply slip to the study team. Potential participants will be contacted by study team researchers to arrange a short screening/recruitment interview, either by telephone or face-to-face.

### Eligibility criteria

Clusters will be family units with at least one young child with an ID. For each cluster, a primary parent/caregiver will be recruited to the study. Subsequently, a secondary family caregiver may be recruited to the study.

- The identified child with ID must meet the following criteria:
  - Inclusion criteria
    - Aged 18 months–5 years (up to the day before the child’s 6th birthday)
    - An administrative label of any severity of ID (learning disability/learning difficulties in UK terminology), referring to identification of the child within the education, health or social care systems as having ID or as eligible for receipt of specialist ID services or diagnoses indicating the presence of ID for younger children (e.g. ‘global developmental delay’) and has a standard score on the Vineland Adaptive Behaviour Scales [18] composite score of < 80 (allowing for measurement error but still indicating significant developmental delay) at the time of the screening interview.
- Exclusion criteria
  - Currently placed in a 24 h residential placement.
  - Currently placed in a foster placement due to end before the 12 month post-randomisation follow-up data collection point.
  - Has current child protection concerns identified.
- The family unit and participants/family caregivers must meet the following criteria:
  - Inclusion criteria
    - A biological, step, adoptive, or foster (if placement is currently planned to extend to 12 months follow-up) parent or adult family caregiver including older siblings, grandparents or other family members who live in the family home.
    - Primary caregiver is available to attend the E-PAtS intervention.
    - Aged ≥ 18 years old.
    - Sufficient level of English language enabling (verbal) completion of outcome measures.
  - Exclusion criteria
    - Enrolled in a group or individually delivered parenting programme outside of the study at baseline (primary family caregiver only).
    - Enrolled in a programme of personal psychological therapeutic support at baseline.
    - Any parent in the family has already participated in an E-PAtS intervention.
    - The family are recognised to be in a state of current crisis and unable to cope/a score of 9 or 10 on the 10-point Brief Family Distress Scale [19] (assessed by primary family caregiver report only). Families in a current state of crisis present with needs that cannot be addressed in a proactive programme and require urgent case management; alternative forms of support will be recommended to families in crisis.

### Study flowchart

**Fig. 1:**
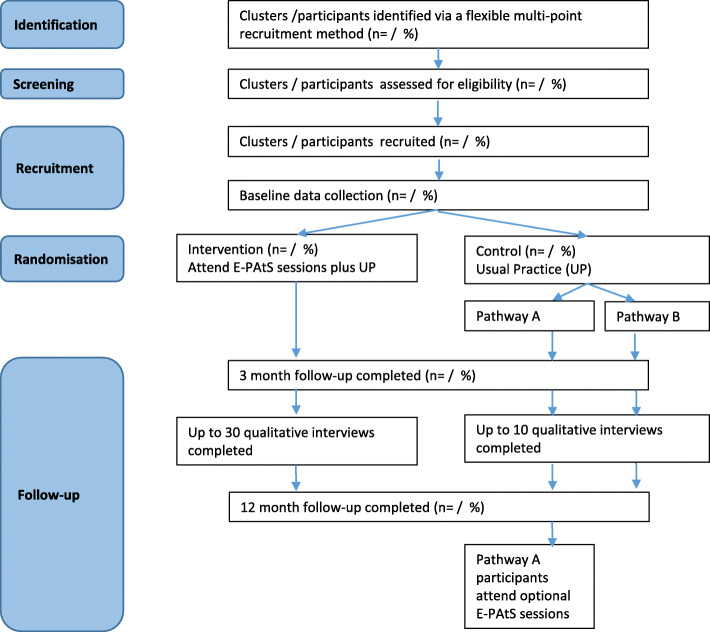
Study flowchart

Figure 1 illustrates the study flowchart.

### Intervention

E-PAtS is a manualised programme for family caregivers of children with ID and will be provided in addition to usual practice (UP).

#### Intervention

The E-PAtS programme comprises (1) an individual preparation interview for family caregivers with the co-facilitator or a representative from their organisation, (2) 8 (typically weekly) 2.5 hourly group sessions, and (3) a personalised workbook and associated resources.

#### Preparation interview

Each family caregiver will attend an individual, supportive, preparatory interview with a co-facilitator or representative of the organisation to enable family caregivers to plan for the attendance, to ensure the group is an appropriate fit to their needs and to resolve any potential barriers, with the ultimate aim to encourage and support attendance and engagement.

#### Group sessions

The group sessions are delivered at times of day determined by the service provider organisation in accordance with the needs and preferences of participating families and may be delivered in a range of community settings including child development centres, community centres and church halls. The content of each session provides an overview of each subject area and focuses on supporting family caregiver wellbeing and parenting behaviour in the context of raising a young child with ID. It provides targeted support and resources to family caregivers to support future engagement with other professional services and systems of social support. The content is evidence-based and utilises theoretical and practical considerations to empower family caregivers and activate improved patterns of family interaction (following the developmental systems model for early intervention [9]). Session content is delivered via presentation and structured exercises but primarily through group discussion. See Table 1 for a summary of each session content.

**Table 1:** E-PAtS group sessions: content overview

Session	Content overview	Specific content
1	**Getting started** Emotional and wellbeing needs of parents/caregivers together with the development of a family system of support.	• Introduction to the programme. • Establishes group process. • Provide advice and strategies to support access to professional services and financial supports for families and their children.
2	**Looking after you and your child** As session 1.	• Discuss emotional vulnerabilities and needs of caregivers of children with ID. Provide advice and strategies to support service access. • Empower parents/caregivers to develop self-management and social support systems and build resilience over the long term.
3	**Sleep** Supporting caregiver knowledge and confidence in responding to child-focussed areas of difficulty that are also associated with poor outcomes for caregivers and families of young children with ID.	• Facilitate supportive dialogues, provide advice and identify strategies to support caregivers to help their child sleep. Consideration of caregiver wellbeing within this context.
4	**Interaction and communication** As session 3.	• Facilitate supportive dialogues, provide advice and strategies to help children acquire effective functional communication. Consideration of caregiver wellbeing within this context.
5	**Fostering life skills through active development** As session 3.	• Facilitate supportive dialogues, provide advice and strategies for development of a range of children’s adaptive skills. Consideration of caregiver wellbeing within this context.
6	**Responding to challenges (1)** As session 3.	• Reinforce previous session content. • Facilitate supportive dialogues and provide additional curriculum to help caregivers prevent and address challenging behaviour currently displayed by their child or that they may be at risk of developing in future. Consideration of caregiver wellbeing within this context.
7	**Responding to challenges (2)** As session 3.	• Reinforce previous session content. • Facilitate supportive dialogues, and provide additional curriculum to help caregivers prevent and address challenging behaviour currently displayed by their child or that they may be at risk of developing in future. Consideration of caregiver wellbeing within this context.
8	**Bringing it all together** Final session to bring together content from all previous sessions and to support the application of programme learning.	• Further expansion of previous content in relation to support in relation to both building systems of family support and safeguarding the emotional wellbeing of parents/caregivers. Consideration of caregiver wellbeing within this context.

The E-PAtS group process aims to create an emotionally and socially supportive setting that encourages engagement and supports the wellbeing needs of family caregivers. Both working with peers who are experiencing similar challenges and difficulties and being supported by a facilitator who is also a caregiver provide emotional validation and inspiration to group members. Presentation of materials and exercises, via a combination of oral and video presentations, group discussion and in-vivo exercises are also designed to support family caregiver engagement, identify their particular needs and strengths and empower them to build upon these.

The content of each session is focused to the needs and circumstances of the attending family caregivers. Prior to delivery of each programme, facilitators are required to make localised adaptions to programme materials (e.g. information provided about current and local financial and service supports). Facilitators are also trained to respond to the specific needs of individual group members during delivery of each session (e.g. citing examples and strategies that are aligned with the presenting needs and circumstances of family caregivers who are in attendance and their children). Family caregivers are given opportunities to rehearse and develop strategies and skills within sessions but not assigned tasks to complete between sessions. This is based on the assumption that participants will likely present with a range of different needs and circumstances and are likely to need to develop family support systems and personal resource as a pre-requisite to implementing self-management and child-focused strategies outside of the session. This may be possible for some participants within the time-frame of programme delivery but more typically is predicted to occur following programme completion.

Family caregivers are encouraged to attend all sessions whether or not they or their child is currently displaying a difficulty in the topic area. This recommendation reflects the fact that caregivers and children with ID are at increased risk of experiencing difficulties across all topic areas sometime in their development, but that this could be reduced through early intervention and proactive support. In addition, it is considered that participating caregivers will contribute towards the group process mechanisms, with the potential to support other group members in relation to one or more of the curriculum areas and that this may have potential benefits for both the caregiver in question and other group members. However, the structuring of the E-PAtS curriculum is arranged to allow some flexibility in attendance to further increase engagement opportunities; key themes (especially parent wellbeing) are repeated throughout all sessions.

#### Personalised workbook

All family caregivers are provided with a workbook to accompany the programme. The workbook contains additional materials, tools and signposting resources in relation to each content area. The workbook is built around a ‘person-centred profile’ detailing the specific support needs for each family’s child. By completing the workbook throughout the programme, families are empowered to create a resource, based on their knowledge and experience, combined with evidence-based practices to inform broader systems of family and child support in the future. The workbook also allows information and learning from the programme to be shared with other family members who are unable to attend sessions directly, contributing towards engagement with fathers and other family caregivers and the development of a shared, collaborative approach for supporting children.

#### Engagement with secondary caregivers

Participation of fathers in group-based parent training occurs at low rates [20]. Engaging with secondary caregivers (including fathers) is an important part of the E-PAtS programme which aims (though does not necessitate) to engage with two family caregivers. The involvement of secondary caregivers is, therefore, explicitly targeted in the programme and a collaborative, shared/co-parenting approach to supporting children is promoted to family caregivers throughout the sessions and is supported via the pre-programme interview and workbook resources.

#### E-PAtS facilitators

The intervention will be co-delivered by a health or social care professional facilitator (including education professionals) and a caregiver facilitator dyad. All facilitators complete a 5-day manualised training programme comprising 1.5 days of teaching in relation to the evidence base, theory and ways of working that underpin E-PAtS; 1.5 days teaching regarding the programme curriculum for E-PAtS; and 2 days of tutoring practice-based demonstration regarding curriculum delivery, group process and co-delivery of E-PAtS. Facilitators need to be able to demonstrate necessary skills and understanding of E-PAtS during the final training session, prior to implementation, and receive a period of supervised practice (between 2 and 3 supervision meetings with the E-PAtS programme trainer during the first facilitation of a programme). Each service provider organisation will be provided with an implementation manual detailing practical elements that the service provider organisation and facilitators need to consider in order to deliver E-PAtS (e.g. role profiles for facilitators, practical suggestions about location set-up and all additional resources required to deliver E-PAtS).

Each service provider organisation will be provided with an implementation manual detailing practical elements that the service provider organisation and facilitators need to deliver E-PAtS (e.g. role profiles for facilitators, practical suggestions about location set-up, and all additional resources required to deliver E-PAtS).

### Usual practice/comparator

The comparator intervention will be usual practice (UP) with optional waitlist E-PAtS. UP includes any service (mainstream and specialised) provided to families and their children with ID as a part of an education, health and care plan (or equivalent outside of England) or via any other mechanism. Children with ID and their families could receive a wide variety of care and support from health, social and education sectors and the third sector depending on their needs. UP may vary by function (e.g. parent support, intervention for the child) and/or by the main recipient (the parent, the child with ID, the whole family). UP may include parenting support or psychological therapy for psychosocial health, but we will not recruit primary caregivers already receiving a recognisable parenting programme intervention or a psychological therapy for mental health problems, at the time of baseline assessments (see Exclusion criteria). UP will be recorded in the study in interviews with family carers and through service use data. In addition, a survey of parents of young children with ID in the UK will be carried out (estimated *n* = 500+) to record their recent use of early years and early intervention services. These data will enable us to assess the families’ level of access to interventions and describe the difference in content, delivery and value between usual practice and E-PAtS. This information will inform a later definitive trial and other future research.

### Retention strategy

To maintain engagement, encourage retention and to thank family caregivers for their time, primary carers will be provided with a £10 high street voucher when contacted to complete follow-up data collection, as has previously been shown to be effective [21]. Contact details will be collected during recruitment, and participants will be reminded by email and text message when a data collection follow-up is due and to complete questionnaires when posted. Participants will also receive a study newsletter at approximately 9–10 months post-randomisation to maintain participant engagement. Participants will be offered three methods of data collection: via telephone, postal or face-to-face at a convenient location. For non-responding participants, a minimum dataset (consisting of 3 prioritised outcome measures (Warwick Edinburgh Well-Being Scale, EQ-5D and Parenting Sense of Competence Scale) aligning with the intervention logic model and taking into consideration participant burden) will be offered to reduce participant burden and maximise follow-up rates.

### Sample size calculation

A total of 64 families (32 families in the usual practice (UP) arm, 32 in the intervention arm) will be recruited. As this is a feasibility study, and the purpose is to provide estimates of key parameters for a future trial rather than to power the current study to detect statistically significant differences, a formal a priori power calculation will not be conducted [22]. However, recruiting 64 families will provide a certain level of precision around a 95% confidence interval (CI). While the sample size is based on families, outcome data will be collected for individual parents. Parents within the same family will be randomised to the same arm, making this a cluster feasibility study with randomisation.

### Outcomes—SPIRIT figure

The following primary outcomes will be measured and used to inform the decision to progress to a definitive trial:

- Recruitment rates and effectiveness of recruitment pathways
- Study retention rates
- Adherence to the E-PAtS programme
- Fidelity of E-PAtS programme delivery
- Service provider organisation recruitment rates and willingness to participate in feasibility and definitive trial.
- Assessment of the barriers and facilitating factors for recruitment, engagement and intervention delivery from the perspective of all stakeholders
- Measurement of usual practice
- Acceptability of collecting and analysing routinely collected data within a definitive trial.

The feasibility of using a range of established outcome measures, proposed to test the intervention in a main trial, will be assessed. This study is not designed to test effectiveness; the acceptability of individual proposed outcome measures (via completion rates, quality of completion and qualitative data) will inform the selection of outcome measures for a definitive trial. The proposed outcome measures will include those for individual family members, sub-system relationships and overall family functioning. Proposed outcomes have been chosen based on experience in research with families of young children with ID including the total measurement load family caregivers have been willing to bear, brevity but with good psychometric properties and potential comparisons with national datasets (e.g. Millennium Cohort Study) to provide context for the meaning of scores obtained. All proposed outcome measures are administered to the family caregiver. Please see Table 2 for details and timings of all proposed outcome measures for a definitive trial (SPIRIT figure) and Additional file 1 for the SPIRIT checklist.

**Table 2:** Participant timeline (SPIRIT figure): schedule of enrolment, interventions and assessments

			Study period				
	Target of outcome (F = family caregiver, C = child)	Screening	Baseline	Randomisation	Follow-up		
Timepoint			Up to 8 weeks prior to randomisation	Up to 5 weeks prior to intervention	3 months post-randomisation	3–9 months post-randomisation	12 months post-randomisation
Enrolment:							
Consent for eligibility	F	X					
Eligibility screen	F	X					
Vineland Adaptive Behaviour Scales (VABS) (3rd) [18]. FULL	C	X					
Brief Family Distress Scale	F	X					
Informed consent	F		X				
Contacts data	F						
Randomisation allocation	N/A			X			
Assessments:							
Demographic data	F						
Warwick-Edinburgh Mental Well-Being Scale [23]	F		X		X		X
Hospital Anxiety and Depression scale [24]	F		X		X		X
EQ-5D-5L [25]	F		X		X		X
Brief COPE [26]	F		X		X		X
Child Behaviour Checklist (CBCL) [27]	C		X		X		X
Paediatric Quality of Life InventoryTM Version 4.0 Generic Core Scales [28]	C		X		X		X
Happiness of relationship scale [29]	F		X		X		X
Family APGAR scale [30]	F		X		X		X
Strengths and Difficulties Questionnaire [31]	F		X		X		X
Sibling Relationship Questionnaire (revised) (where relevant) [32]	C		X		X		X
Family Support Scale [33]	F		X		X		X
Five Minute Speech Sample [34]	F and C		X		X		X
Parenting Sense of Competence Scale (7 items) [35]	F		X		X		X
Positive Gains Scale [36]	F		X		X		X
Disagreement over issues related to child [29], co-parenting [37]	F		X		X		X
Child-parent relationship scale [38]	F and C		X		X		X
Parent activities/involvement index	F and C		X		X		X
Group Cohesion Scale (8 items) [39]	F				X		
Client Service Receipt Inventory [40]	F & C		X		X		X
Vineland Adaptive Behaviour Scales (VABS) (3rd) [18]. Brief.	C						X
Participant views on use of routine collected data in future trial	F						X
Process evaluation-participant interviews	F					X	
Process evaluation—facilitator interviews	N/A					X	
Process evaluation—service provider organisation interviews	N/A					X	

### Participant flow/procedure

Figure 1 illustrates the study flowchart.

### Data collection methods

#### Participant screening

A screening interview will be conducted either via telephone or face-to-face with a study team research assistant (RA). Study processes, in particular the screening process, will be fully explained, and family caregivers will be provided a participant information sheet by email or post. Written consent or verbal consent will be obtained in face-to-face or telephone screening interviews respectively. Screening measures will be taken to establish eligibility, including the Vineland Adaptive Behaviour Scales (VABS) [18] and the Brief Family Distress Scale [19]. Scoring of the VABS will be conducted following the screening visit by the RA and quality checked by a trained member of the study team. The family caregiver will be informed of their eligibility and if applicable, a recruitment interview arranged.

#### Recruitment and consent

A recruitment interview will be conducted either via telephone or face-to-face with a study team RA. Participants will have been provided with the participant information sheet (see ‘Participant Screening’ section) and given sufficient time to consider the information prior to the interview. All study processes will be explained in detail, including randomisation and burden for the participant. Written consent or verbal consent will be obtained in face-to-face or telephone interviews respectively. The following data collection forms will be completed: a participant’s contact form including multiple methods of contact (address, telephone, email address) to minimise loss to follow-up; preferences for follow-up data collection (face-to-face interview completion, telephone-based completion or postal questionnaires); preferences for choice of study pathway (participants randomised to the control group who choose pathway A will be invited to attend E-PAtS 12 months post-randomisation and participants who choose pathway B will not be invited to an E-PAtS course); baseline questionnaire including baseline demographics and outcome measures.

#### Frequency and duration of follow-up

Data will be collected at 3 months post-randomisation and 12 months post-randomisation. Participants will be contacted by RAs to complete the 5 min speech sample at 3 months post-randomisation and brief VABS at 12 months post-randomisation, either by telephone or face-to-face. All remaining outcome measures (see Table 2) will be collected via postal questionnaire, telephone interview by the RA or face-to-face interview with the RA, depending on the participants’ preference at baseline and whether participants have requested an alternative means of data collection subsequently. To reduce the risk of bias, RAs will read questions from the questionnaire directly, remain blind to the participants’ allocation and will ask participants not to reveal their allocation. If allocation is revealed, this will be noted. Intervention participants who requested telephone or face-to-face data collection at the 3 months post-randomisation follow-up will be contacted by the study/data manager to complete the group cohesion scale to prevent unblinding to the RA in these instances. Programme attendance will be collected by the facilitators and supplied to the research team directly.

#### Randomisation/sequence generation

The study is a 2-arm, cluster randomised controlled trial. Clusters will be families with a child with ID, and up to 2 participants will be recruited per cluster. Families will be randomised post recruitment and completion of all baseline measures. Families will be randomised, using randomly permuted blocks, stratified by study site and choice of study pathway (pathway A or B) and an equal allocation 1:1 ratio to E-PAtS in addition to usual practice (UP) or UP alone. The study manager/data manager will conduct randomisation and will inform participants, and the service provider organisation, of their allocation by telephone. RAs at site, responsible for collecting follow-up data, and all remaining study team members (including the trial statistician) will remain blind to participants’ allocation.

### Process evaluation

MRC guidance [9] will be used as a framework for the process evaluation to describe implementation processes, refine the intervention logic model through examining intervention mechanisms and consider the role of context in shaping intervention implementation and mechanisms. The process evaluation will employ a mixed methods approach and will focus on the study primary objectives. Quantitative methods including attendance logs and intervention self-report checklists, completed by facilitators, and intervention video/audio recordings will be used to assess recruitment rates/patterns, attendance and intervention fidelity, reach and adherence. Using a pre-defined checklist, fidelity will be assessed by determining the proportion of key messages and activities which are completed as intended in each session. Qualitative interviews with facilitators, service provider organisations and family caregivers will examine implementation processes, intervention mechanisms, and the role of contextual factors and interrogate patterns in the quantitative data, as well as inform assessment of the feasibility of implementing E-PAtS within a definitive trial.

### Data management and security

All data will be checked visually on receipt by the study administrator, data manager or study manager. Study management data will be entered on to a MS Access Database by the study manager or study administrator. Study data will be entered on to paper questionnaires/case report forms either by the participants, facilitators or by RAs at site and returned to the study team by post or couriered securely. RAs will be trained in Good Clinical Practice (GCP) and study-specific processes. Participant data will be pseudonymised and entered manually onto a secure, password-protected Microsoft SQL database by the study administrator (SA) and data queries noted. Ten percent of all data will be quality checked and all data queries actioned by the data manager. Any key data queries will be taken to the study management group or steering committee as appropriate. Finally, data will be checked during data cleaning using SPSS syntax for validations and missing data. Hard copies of personally identifiable and research data will be held separately and securely in a locked cupboard, with access limited to essential research team members.

Qualitative interview and observation recordings will be recorded on encrypted audio-recorders/video recorders and stored on password-protected computers at site. Recordings will be securely transferred to the study team at the Centre for Trials Research by Fastfile or courier. All files will be encrypted, and transcripts will be fully pseudonymised prior to analysis. All qualitative interviews will be audio-recorded, transcribed fully, and pseudonymised for analysis. Computer software (NVivo) will be used to manage the qualitative data and transcripts. Data security and confidentiality will be ensured, in line with GDPR. A data management plan will be completed and adhered to. Only the trial team will have access to the final study dataset.

### Statistical methods/analysis plan

This protocol paper follows SPIRIT guidelines, and the analysis and reporting of this pragmatic randomised controlled trial will be in accordance with CONSORT (Consolidated Standards of Reporting Trials) guidelines. Significance tests will not be reported as the E-PAtS feasibility RCT is not powered to test hypotheses. The majority of outcome analysis will be descriptive in nature. Continuous data will be reported as means and standard deviations or medians and interquartile ranges, as appropriate. Categorical data will be reported as frequencies and proportions. Feasibility outcomes will be estimated with their associated 95% confidence intervals. The main preliminary analyses of outcomes will be intention-to-treat-based, accounting for clustering (groups in intervention arm, family caregivers in families) using multilevel models. Single caregiver families will be included as a cluster of size 1*.* The analysis of the proposed primary outcome for a definitive trial will examine mean WEMWBS scores between arms at 12 months post-randomisation, with baseline WEMWBS scores included as a covariate. The analysis will also adjust for randomisation factors. Remaining proposed outcomes for a definitive trial (including outcomes at 3 months post-randomisation) will be analysed similarly, with appropriate multilevel regression models. An exploratory complier average causal effect analysis will also be conducted, focused on family caregivers who complete the E-PAtS programme (see earlier definitions of completion/adherence). Results from all regression models will be reported using point estimates and 95% confidence intervals.

### Health economic methods/analysis plan

The feasibility study will also be used as a vehicle to: (i) evaluate the performance of client service receipt inventories (administered at baseline and at 3 months and 12 months post-randomisation) in collecting resource utilisation; (ii) assess the availability of routine health and social data sources that can be used to complement and validate self-reported resource utilisation data; (iii) identify appropriate sources of unit costs for potential resource consequences and assess how much primary costing research will be required for a definitive trial-based economic evaluation; and (iv) identify the best possible way of expressing the cost-effectiveness of the E-PAtS programme using preference-based approaches.

### Qualitative methods/analysis plan

Thematic analysis [41] will be used to analyse each group of interviews (service provider organisations, facilitators, family caregivers) separately and independently followed by qualitative synthesis across all interviews to provide an over-arching synthesis of family caregivers’ experiences and perceptions related to the study objectives. A triangulation exercise will be conducted combining qualitative and quantitative data analysis results.

A full statistical analysis plan will be written by the statistician and qualitative researcher and approved by the study management group and study steering committee prior to any analysis taking place.

### Progression criteria for a definitive trial

The following criteria will inform the decision to progress to a definitive trial, with consideration to issues that may have affected meeting any of these criteria and steps that can be taken to overcome these issues within a full trial:

- Recruitment of families—50% of families approached, and who are eligible, consent to the study.
- Rate of recruitment—the target sample of 64 families is achieved within the study recruitment period.
- Randomisation feasibility—10–16 families are recruited in a local area of the E-PAtS service provider organisation to allow randomisation and a maximum of 8 families per E-PAtS group.
- Study retention—75% of primary caregivers are retained for follow-up at 12 month data collection point.
- Adherence—70% of primary caregivers and 40% of recruited secondary caregivers adhere to the E-PAtS programme.
- Fidelity—70% of E-PAtS curriculum components are rated as partially or fully present in all recorded group sessions available for analysis.
- Service provider organisation willingness to participate in a definitive trial—a sufficient number of training providers indicate a willingness to take part in a definitive trial.
- E-PAtS intervention is sufficiently different to usual practice
- Study steering committee consensus recommends progression to a definitive trial.

### Adverse event reporting

There are no expected adverse events related to the intervention or research procedures; the University of Warwick Humanities and Social Sciences Research Ethics Committee has approved that adverse events should not be reported for this study. Any families who are considered in crisis at screening will be referred for urgent case management, following site-specific protocols. If throughout the duration of the study, a member of the study team becomes concerned regarding the wellbeing or safety of a study participant or their child, study staff will follow study and site-specific protocols for dealing with harm.

### Auditing

No independent audits are planned.

### Study governance

Ethical approval for this study was given by the University of Warwick Humanities and Social Sciences Research Ethics Committee on the 14th of December 2017, reference number 30/17-18. Any protocol amendments will be approved by the University of Warwick Humanities and Social Sciences Research Ethics Committee. A study steering committee (SSC) will meet approximately every 6 months to provide study oversight. The SSC comprises two independent academic social workers (one of whom is the Chair), an independent statistician and a lay representative.

### Confidentiality

All data will be kept for 15 years in line with Cardiff University’s Research Governance Framework Regulations for clinical research. Electronic data will be stored confidentially on password-protected servers maintained on University networks. All hard copy forms will be stored in locked filing cabinets. For participant interviews, all audio files will be recorded on encrypted audio-recorders and securely held in password-protected servers maintained on University networks. Audio files will be transcribed and pseudonymised using University-approved transcription companies. No identifiable data will be published.

### Dissemination policy

A publication plan and dissemination policy will be written. The study results will be disseminated in full and with a lay summary on the Centre for Trials Research (CTR), University of Warwick and University of Kent websites and a summary of the results will be disseminated to all participants. Any data requests should be made to the CTR. The CTR is a signatory of AllTrials and aims to make its research data available wherever possible.

### Public involvement

A Project Advisory Group (PAG) of family caregivers of young children with ID will be established to offer recommendations for participant facing materials including outcome measures, offer strategic advice on engaging families, act as ambassadors for the study, create communication pathways with family caregivers of young children with ID and parent networks, to contribute to the interpretation of the feasibility study findings and assist in the co-production of dissemination outputs for family caregivers. A face-to face meeting will take place at least twice, supplemented by ad hoc contact via email at study milestones. The SSC will include an independent lay representative with experience of parenting of child with ID.

## Discussion

Despite the high level of need for parents and children with ID and the drive and commitment of the NHS to implement early years parenting programmes, as identified in the Transforming Care (TC) programme [33], there is currently a significant gap in the evidence base and availability of suitable programmes. The E-PAtS logic model directly addresses this gap, and its aims are specifically aligned to reduce inequalities in service provision and target health-focused outcomes for family caregivers and children with ID. The results of this feasibility study will contribute to the evidence base on improving outcomes for children with ID and their family caregivers. In addition, the findings from this feasibility study will determine the progression to a definitive trial to assess the effectiveness and cost-effectiveness of the E-PAtS intervention and will inform parameters of study design specifically recruitment processes, methods of data collection and choice of outcome measures.

### Study status

Current protocol: version 1.2 31/01/18. Recruitment start date: 26 March 2018. Approximate recruitment end date: 31 May 2019

## Supplementary information

**Additional file 1.** SPIRIT Checklist

## Data Availability

Not applicable—protocol paper.

## Abbreviations

CONSORT: Consolidated Standards of Reporting Trials
CTR: Centre for Trials Research
E-PAtS: Early Positive Approaches to Support
GCP: Good Clinical Practice
IQ: Intelligence quotient
ID: Intellectual disability
NHS: National Health Service
NICE: The National Institute for Health and Care Excellence
PAG: Project Advisory Group
RA: Research assistant
RCT: Randomised controlled trial
SA: Study administrator
SPIRIT: Standard protocol items: recommendations for interventional trials
SSC: Study steering committee
TC: Transforming Care
UP: Usual practice
VABS: Vineland Adaptive Behaviour Scales

## References

[1] Hatton C, Emerson E, Glover G, Robertson R, Baines S, Christie A (2014). People with learning disabilities in England 2013.

[2] Emerson C, Hatton C (2007). Mental health of children and adolescents with intellectual disabilities in Britain. Br J Psychiatry.

[3] Totsika V, Hastings RP, Emerson E, Lancaster GA, Berridge DM (2011). A population-based investigation of behavioural and emotional problems and maternal mental health: associations with autism and intellectual disability. J Child Psychol Psychiatry.

[4] Totsika V, Hastings RP, Emerson E, Berridge DM, Lancaster GA (2011). (2011). Behaviour problems at five years of age and maternal mental health in autism and intellectual disability. J Abnorm Child Psychol.

[5] Hastings RP (2016). Do children with intellectual and developmental disabilities have a negative impact on other family members? The case for rejecting a negative narrative. Int Rev Res Dev Disabil.

[6] Hastings RP, Daley D, Burns C, Beck A (2006). Maternal distress and expressed emotion: cross-sectional and longitudinal relationships with behavior problems of children with intellectual disabilities. Am J Ment Retard.

[7] Cridland EK, Jones SC, Magee CA, Caputi P (2014). Family-focused autism spectrum disorder research: a review of the utility of family systems approaches. Autism..

[8] Trivette CM, Dunst CJ, Hamby DW (2010). Influences of family-systems intervention practices on parent-child interactions and child development. Topics in Early Childhood Special Education..

[9] Toms G, Totsika V, Hastings RP, Healy H (2015). Access to services by children with intellectual disability and mental health problems: population-based evidence from the UK. J Intellect Dev Disabil.

[10] Totsika V, Hastings RP, Vagenas D, Emerson E (2014). Parenting and the behavior problems of young children with an intellectual disability: concurrent and longitudinal relationships in a population-based study. American Journal on Intellectual and Developmental Disabilities..

[11] National Institute for Health and Care Excellence (2016). Mental health problems in people with learning disabilities: prevention, assessment, and management. NICE Guideline NG54.

[12] Tellegen CL, Sanders MR (2013). Stepping Stones Triple P–Positive Parenting Program for children with disability: a systematic review and meta-analysis. Research in Developmental Disabilities..

[13] Durand VM, Hieneman M, Clarke S, Wang M, Rinaldi M (2013). Positive family intervention for severe challenging behaviour: a multisite randomized clinical trial. J Positive Behav Interventions.

[14] Gore NJ, Hastings RP, Brady S (2014). Early intervention for children with learning disabilities: making use of what we know. Tizard Learn Disabil Rev.

[15] Guralnick MJ (2001). A developmental systems model for early intervention. Infants and Young Children..

[16] ADASS LGA, England NHS (2015). Supporting people with a learning disability and/or autism who display behaviour that challenges, including those with a mental health condition: service model for commissioners of health and social care services. London.

[17] Scottish Government (2012). National Parenting Strategy: making a positive difference to children and young people through parenting.

[18] Sparrow SS, Cicchetti DV, Saulnier CA (2016). Vineland Adaptive Behavior Scales, survey forms manual (3rd ed.).

[19] Weiss JA, Lunsky Y (2011). The Brief Family Distress Scale: a measure of crisis in caregivers of individuals with autism spectrum disorder. J Child Fam Stud.

[20] Lindsay G, Strand S (2013). Evaluation of the national rollout of parenting programmes across England: the parenting early intervention programme (PEIP). BMC Public Health..

[21] Brueton VC, Tierney JF, Stenning S, Meredith S, Harding S, Nazareth I, Rait G (2014). Strategies to improve retention in randomised trials: a Cochrane systematic review and meta-analysis. BMJ open.

[22] Arain M. What is a pilot or feasibility study? A review of current practice and editorial policy. BMC Med Res Methodol. 2010;10(1):67. 10.1186/1471-2288-10-67 PMC2912920 20637084

[23] Tennant R, Fishwick R, Platt S, Joseph S, Stewart-Brown S (2006). Monitoring positive mental health in Scotland: validating the affectometer 2 scale and developing the Warwick Edinburgh Mental Well-being Scale for the UK.

[24] Zigmond AS, Snaith RP (1983). The hospital anxiety and depression scale. Acta Psychiatrica Scandinavica..

[25] van Hout B, Janssen MF, Feng YS, Kohlmann T, Busschbach J, Golicki D, Lloyd A, Scalone L, Kind P, Pickard AS (2012). Interim scoring for the EQ-5D-5L: mapping the EQ-5D-5L to EQ-5D-3L value sets. Value Health..

[26] Carver CS (1997). You want to measure coping but your protocol’s too long: consider the Brief COPE. International Journal of Behavioral Medicine..

[27] Achenbach TM, Rescorla LA (2001). Manual for the ASEBA preschool forms and profiles.

[28] Varni JW, Seid M, Kurtin PS (2001). PedsQL^TM^ 4.0: reliability and validity of the Pediatric Quality of Life Inventory^TM^ Version 4.0 Generic Core Scales in healthy and patient populations. Med Care.

[29] University of London. UCL Institute of Education. Centre for Longitudinal Studies, Millennium Cohort Study, Wave 2. https://cls.ucl.ac.uk/cls-studies/millennium-cohort-study/.

[30] Smilkstein G (1978). The family APGAR: a proposal for a family function test and its use by physicians. J Fam Pract.

[31] Goodman R (1997). The strengths and difficulties questionnaire: a research note. Journal of Child Psychology and Psychiatry and Allied Disciplines..

[32] Furman W, Buhrmester D (1985). Children’s perceptions of the qualities of sibling relationships. Child Development..

[33] Dunst CJ, Jenkins V, Trivette CM (1984). The family support scale: reliability and validity. J Individ Fam Commun Wellness.

[34] Magana AB, Goldstein MJ, Karno M, Miklowitz DJ, Jenkins J, Falloon IRH (1986). A brief method for assessing expressed emotion in relatives of psychiatric patients. Psychiatr Res.

[35] Johnston C, Mash EJ (1989). A measure of parenting satisfaction and efficacy. Journal of Clinical Child Psychology.

[36] MacDonald EE, Hastings RP, Fitzsimons E (2010). Psychological acceptance mediates the impact of the behaviour problems of children with intellectual disability on fathers’ psychological adjustment. J Appl Res Intellect Disabil.

[37] Feinberg ME, Brown LD, Kan ML (2012). A multi-domain self-report measure of coparenting. Parenting..

[38] Pianta RC (1995). Child-parent relationship scale.

[39] Treadwell T, Lavertue N, Kumar VK, Veeraraghavan V (2001). The group cohesion scale-revised: reliability and validity. Int J Act Method.

[40] Beecham J, Knapp MRJ, Thornicroft GJ, Brewin CR, Wing JK (1992). Costing psychiatric interventions. Measuring mental health needs.

[41] Braun V, Clarke V (2006). Using thematic analysis in psychology. Qualitative Research in Psychology..

